# A review of existing and potential computer user interfaces for modern radiology

**DOI:** 10.1007/s13244-018-0620-7

**Published:** 2018-05-16

**Authors:** Antoine Iannessi, Pierre-Yves Marcy, Olivier Clatz, Anne-Sophie Bertrand, Maki Sugimoto

**Affiliations:** 1 Interventional and Diagnostic Imaging Department, Cancer Center Antoine Lacassagne Nice, Nice, France; 2 Imaging Department, Polyclinique les Fleurs, Ollioules, France; 3 Therapixel, Valbonne, France; 4 International University of Health Welfare, Otawara, Japan

**Keywords:** Computer user interface, Computed tomodensitometry, Virtual reality, Volume rendering, Interventional radiology

## Abstract

**Abstract:**

The digitalization of modern imaging has led radiologists to become very familiar with computers and their user interfaces (UI). New options for display and command offer expanded possibilities, but the mouse and keyboard remain the most commonly utilized, for usability reasons. In this work, we review and discuss different UI and their possible application in radiology. We consider two-dimensional and three-dimensional imaging displays in the context of interventional radiology, and discuss interest in touchscreens, kinetic sensors, eye detection, and augmented or virtual reality. We show that UI design specifically for radiologists is key for future use and adoption of such new interfaces. Next-generation UI must fulfil professional needs, while considering contextual constraints.

**Teaching Points:**

• *The mouse and keyboard remain the most utilized user interfaces for radiologists*.

• *Touchscreen, holographic, kinetic sensors and eye tracking offer new possibilities for interaction*.

• *3D and 2D imaging require specific user interfaces*.

• *Holographic display and augmented reality provide a third dimension to volume imaging*.

• *Good usability is essential for adoption of new user interfaces by radiologists*.

## Introduction

The digitalization of modern imaging facilitates exchange and archiving, and enables the application of advanced image analysis solutions such as computer-aided detection (CAD) for identification of small lesions in several organs. The shift from analog to digital imaging should have led to an increase in efficiency among radiologists by reducing the time for interpretation and image manipulation. This has not been clearly demonstrated, and one limiting factor is represented by what is called the computer user interface. Advances in recent years have enabled the availability of touchscreens and new sensor devices for eye, kinetic or voice commands at low cost, offering expanded possibilities for this human–computer interaction.

### Terminology and concepts

The *user interface* (*UI*), also known as the human–machine interface, is defined as all the mechanisms (hardware or software) that supply information and commands to a user in order to accomplish a specific task within an interactive system. All machines (e.g. cars, phones, hair dryers) have a UI. The computer has a global UI called an operating system, such as Windows or Mac OS X. A web browser has a specific UI, and a web site itself has a specific UI. In practice, the UI is the link between the machine and the operator. In informatics, the UI includes inputs and outputs. Inputs communicate a user’s needs to the machine, and the most common are the keyboard, mouse, touch interface and voice recognition. New sensor devices for eye and kinetic commands that have recently been developed offer greater sophistication at low cost, enhancing the potential of this human–computer interaction [1]. Outputs communicate the results of a computer’s calculation to the user. The most common UI is a display screen, but sound and haptic feedbacks are sometimes used.

**Fig. 1:**
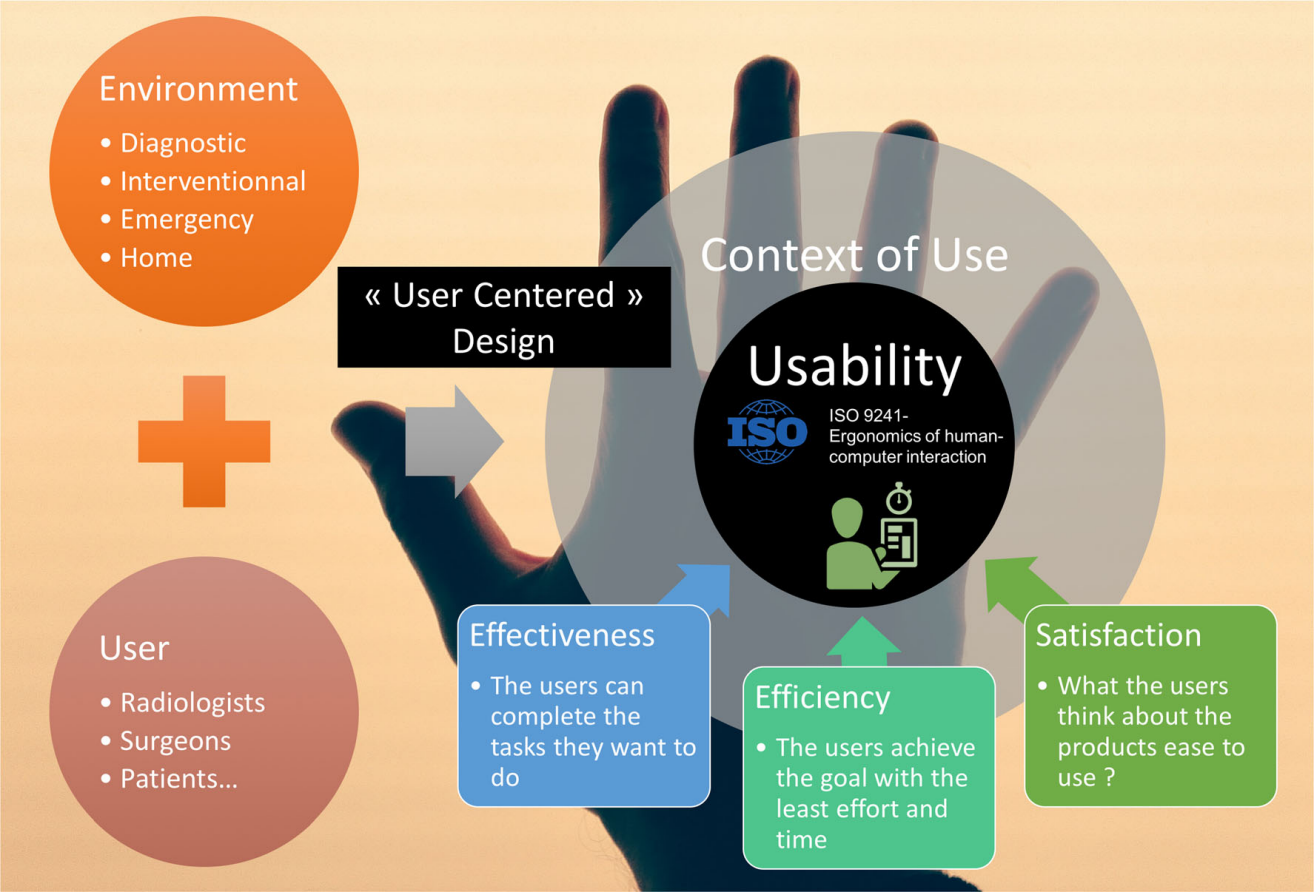
The usability of a computer user interface (CUI) in radiology is evaluated by three indicators. The UI is designed to maximize the usability in a specified context of use. In medical imaging, the usage context can be defined as a user (the reader of the images) inside his environment

The *user experience* (*UX*) is the outcome of the UI. The objective is to provide the best usability to achieve a good UX. This depends on both user specificity and the specific usage context. For these reasons, the UX is evaluated on the basis of both psychology and ergonomics. Ergonomic requirements are defined by International Organization for Standardization (ISO) 9241 regulatory standard, to ensure the operator’s comfort and productivity, preventing stress and accidents (Fig. 1) [2]. Usability is high when efficacy, efficiency and satisfaction are high. Efficacy is the user's ability to complete the planned task. Efficiency is measured by the time to completion. Satisfaction is the user’s subjective evaluation of the comfort of use. The UI needs to be developed and designed specifically for the context of use. This “user-centred design” aims to maximize usability.

### Computed radiology and specific needs

**Fig. 2:**
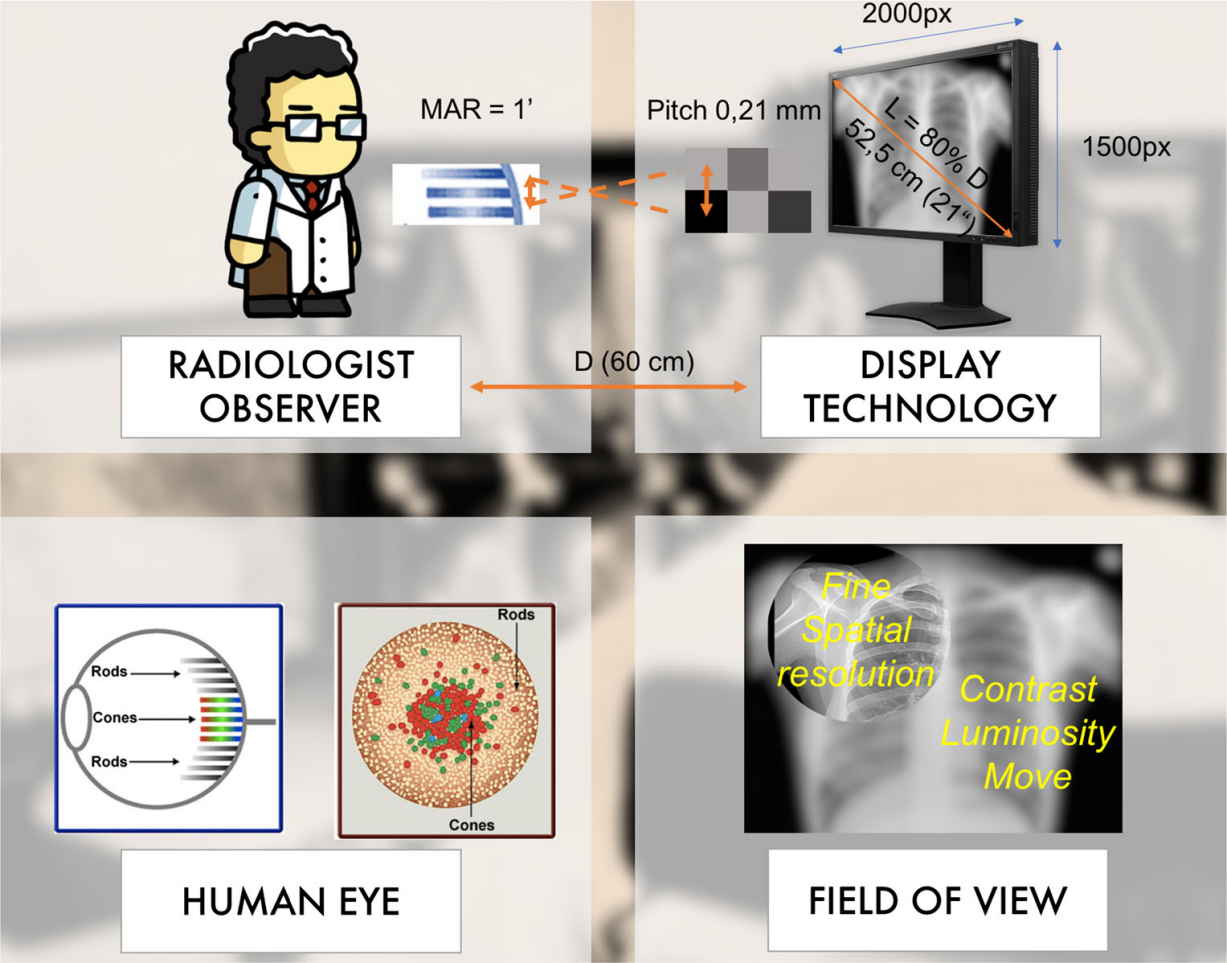
The human–machine interaction is constrained by human and contextual factors. Typically, the display is around 60 cm from the radiologist. At this distance, the field of view is around 50 cm (around 21 in.). Considering the maximum angular resolution of the eye, the display can have maximum pitch of 0.21 mm. This corresponds to a 3-megapixel screen. The human retina contains two types of photoreceptors, rods and cones. The cones are densely packed in a central yellow spot called the “macula” and provide maximum visual acuity. Visual examination of small detail involves focusing light from that detail onto the macula. Peripheral vision and rods are responsible for night vision and motion detection

With regard to medical imaging, the UI is specifically constrained by human and contextual factors [3]. The interaction occurs between a human observer and a display technology (Fig. 2). With respect to human visual ability, the eye is made of cones and rods. The cones are concentrated in the macula at the centre of the field of vision and provide the best spatial resolution. On the periphery of the visual field, the image becomes blurry. The eye's maximum power of discrimination is 0.21 mm. The physiological focal point of the eye is around 60 cm from the viewer. This creates the technical standards for the practice of radiology. The diagonal of the display should be located at 80% of the distance to the eye, which corresponds to a screen of approximately 50 cm (about 21 in.). For this size, the resolution providing a pitch of 0.21 mm is 1500×2000 pixels [4]. Diagnostics are performed using “macular vision”. The radiologist needs to explore the whole image by moving the eye. This justifies the need for pan and zoom tools to study a particular region of interest.

With regard to contextual constraints, we differentiate diagnostic from interventional radiology. Indeed, with regard to the former, the constraints of UX are more about managing the workflow for maximum productivity. One challenge is the integration of different commands and information in a common UI. Regarding the latter, the limit is clearly in maintaining operator sterility.

In this paper, we review and discuss different UI tools available for radiology over time. We also try to provide an outlook for the future and suggestions for improvements.

## Review of the literature

### Interfaces for 2D images

Imaging devices have largely provided two-dimensional images: X-ray planar imaging at the beginning of the twentieth century, and then computed tomography (CT) or magnetic resonance (MR) sliced imaging in the 1970s. Initially, the image was printed like a photo, and there was no machine interaction. Today, if we look at any interpretation room around the world, chances are good that we will find the same setup, combining a chair, a desk and one or more computers with a keyboard, a mouse and a screen. The picture of modern radiology can be understood through the evolution of the computer UI, as the image has become digital and is displayed on computers.

**Fig. 3:**
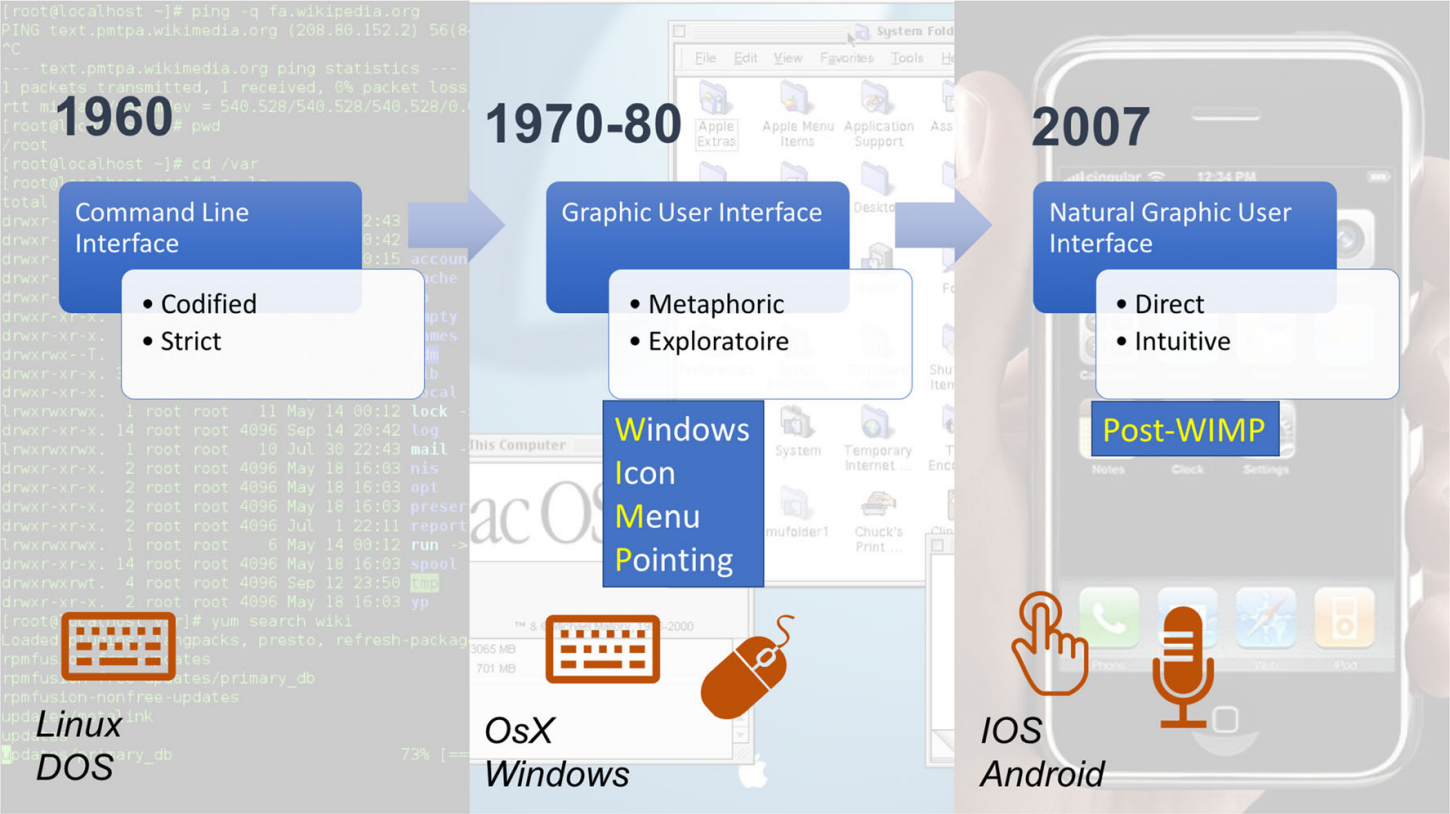
History of common computer user interfaces. The most common user interface (UI) is the graphical UI (GUI) used by operating systems (OS) of popular personal computers in the 1980s. It is designed with a mouse input to point to icons, menus and windows. Recently, new OS with specific interfaces for touchscreens have emerged, known as natural user interfaces (NUI)

In the 1960s, the command-line interface (CLI) was the only way to communicate with computers. The keyboard was the only input, and a strict computer language had to be known to operate the system. In 1966, Douglas Engelbart invented the computer mouse. Together with Xerox, and then Apple's Mac OS X or Microsoft Windows, they participated in developing a graphical user interface (GUI), known as “WIMP” (windows, icons, menus and pointing device) [5], which vastly improved the user experience. This system made computers accessible to everyone, with minimal skill required. Today, the WIMP UI remains nearly unchanged, and it is the most commonly used UI for personal computers. In 2007, the post-WIMP era exploded with the introduction of the “natural user interface (NUI)” using touchscreens and speech recognition introduced by Apple iOS, followed by Google Android, used mainly for tablet personal computers (PC) and smartphones (Fig. 3) [6].

Digital radiology and the current workstation were introduced during the WIMP era. The specific UI was designed with a keyboard and a mouse, and this setup has remained in use for approximately 30 years. Resistance to change and the “chasm” or delay in the new technology adoption curve explains the UI stagnation globally in the field of radiology [7].

However, is there really a better alternative to a mouse and a keyboard? Weiss et al. tried to answer this question, and compared five different setups of IU devices for six different PACS users during a 2-week period [8]. The study did not include post-WIMP UI. The authors concluded that no one device was able to replace the pairing of the keyboard and mouse. The study also revealed that the use of both hands was thought to be a good combination. However, the evaluation focused on image manipulation and did not consider single-handed control needed for microphone use in reporting.

The authors proposed an interesting marker of efficacy for radiologic IU as the highest ratio of “eyes-to-image” versus “eyes-to-interface” device time.

**Fig. 4:**
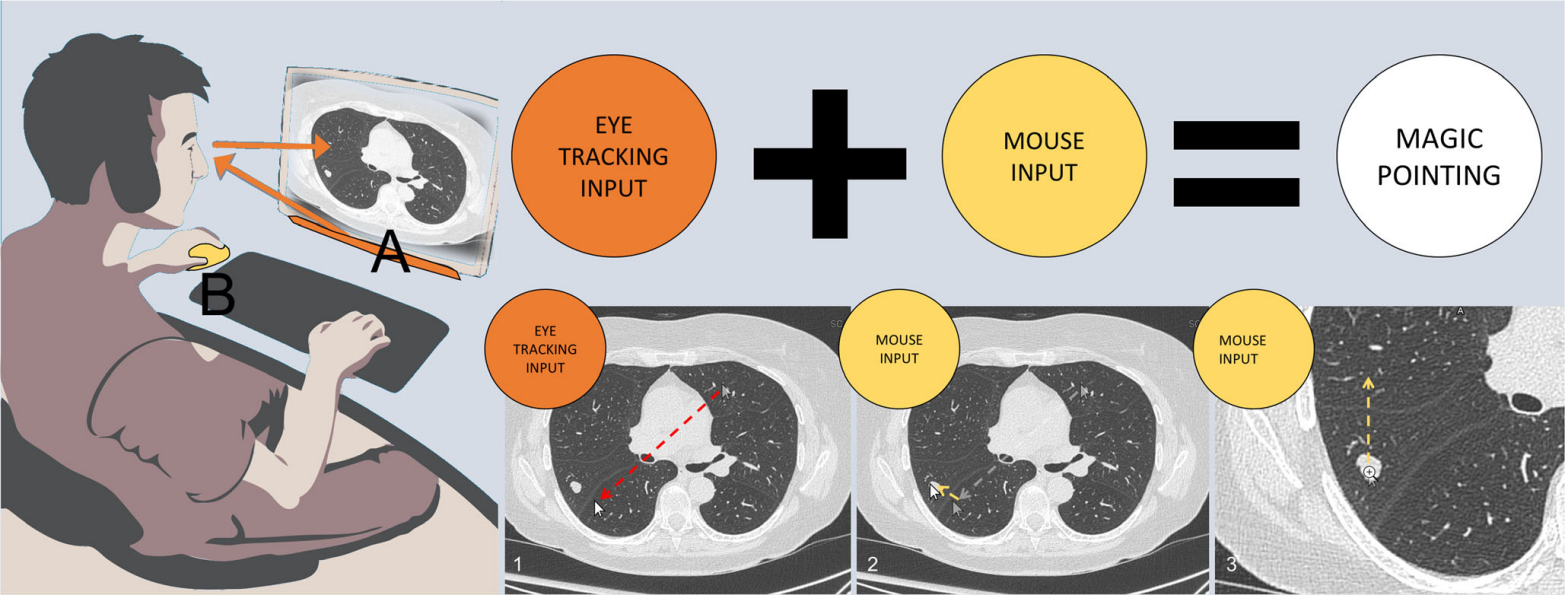
Potential use of manual and gaze input cascaded (MAGIC) pointing for diagnostic radiology. The radiologist is examining lung parenchyma. When he focuses on an anomaly, the eye tracking device automatically moves the pointer around the region of interest. A large amount of mouse movement is eliminated (dotted arrow), and is limited to fine pointing and zooming. This cascade follows the observer's examination, making the interaction more natural

Some solutions for improving the WIMP-UI have been tested. They combine an “eye tracking” technology with the pointing technique [9]. The objective is to eliminate a large portion of cursor movement by warping the cursor to the eye gaze area [10]. Manual pointing is still used for fine image manipulation and selection. Manual and gaze input cascaded (MAGIC) pointing can be adapted to computer operating systems using a single device (Fig. 4).

**Fig. 5:**
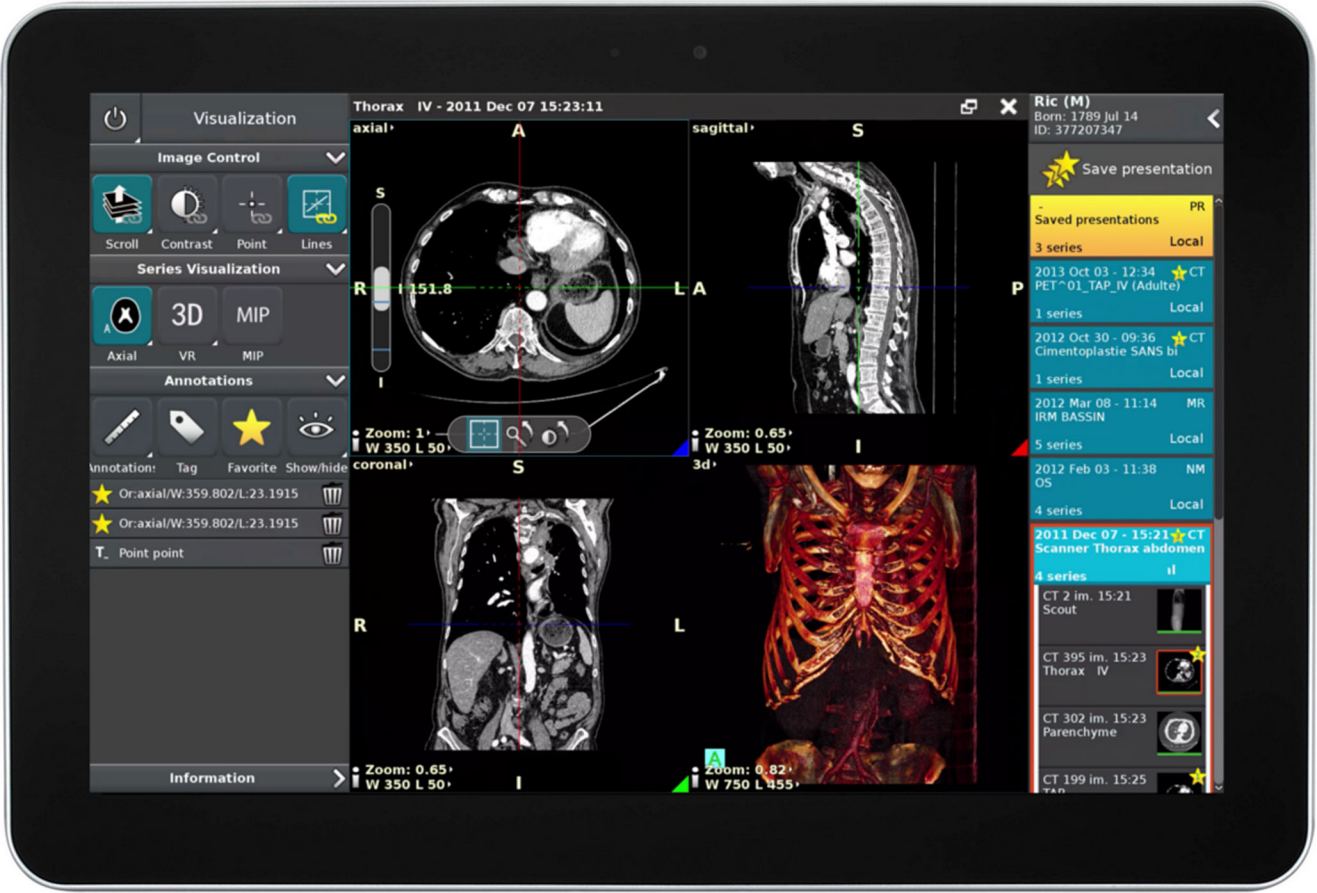
Tactile version of the Anywhere viewer (Therapixel, France). Cloud computing allows powerful post-processing with online PACS

**Fig. 6:**
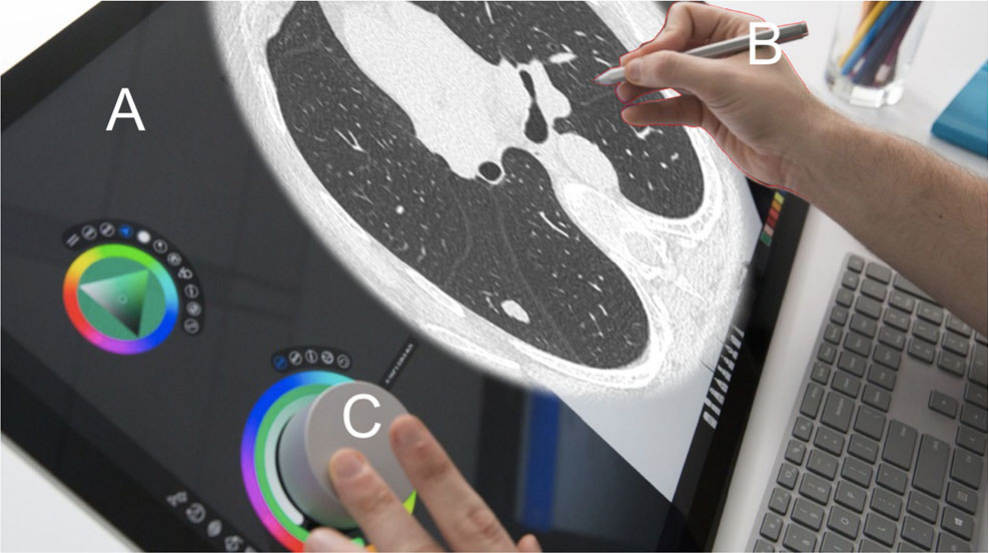
Potential use of Surface Studio^®^ (Microsoft, Redmond, WA, USA). The screen is tactile (**a**). The stylet would be handy for direct measurement and annotation of the image (**b**). The wheel could be used to select functions such as Windows and for scrolling of images (**c**). A properly designed software interface could replace traditional mouse and computer workstations

Regarding post-WIMP UI, and especially touchscreens, there is abundant literature, most of which deals with emergency setup involving non-radiologist readers as well [11]. Indeed, the tablet PC offers greater portability and teleradiology possibilities. Tewes et al. showed no diagnostic difference between the use of high-resolution tablets and PACS reading for interpreting emergency CT scans [12]. However, touchscreen adoption is not evident at the moment, even if full high-definition screens fulfil quality assurance guidelines. Users have found the windowing function less efficient than the mouse, and have also noted screen degradation due to iterative manipulations. Technically, portable tablet size and hardware specifications are not powerful enough for image post-processing. However, cloud computing and streaming can provide processor power similar to a stand-alone workstation (Fig. 5). Their portability makes them more adaptable for teleradiology and non-radiology departments. One possible solution discussed recently is a hybrid type of professional tablet PC for imaging professionals [13]. The interface is designed to enable direct interaction on the screen using a stylet and another wheel device. Microsoft and Dell are currently proposing design solutions for specific use with photo and painting software. These desktops could be used for radiology workstations with a few UX-specific design modifications (Fig. 6).

Interventional radiology is a specific process with specific needs, the most important of which is maintaining the sterility of the operating site while manipulating the images. Ideally, the operation has to be autonomic for at least basic features such as selecting series, reformatting, slicing, and pan and zoom manipulation. Some have proposed taking a mouse or trackpad inside the sterile protected area, or even using a tablet PC to visualize images. However, the most efficient setup in these conditions is touchless interaction [14], which will minimize the risk of contamination.

**Fig. 7:**
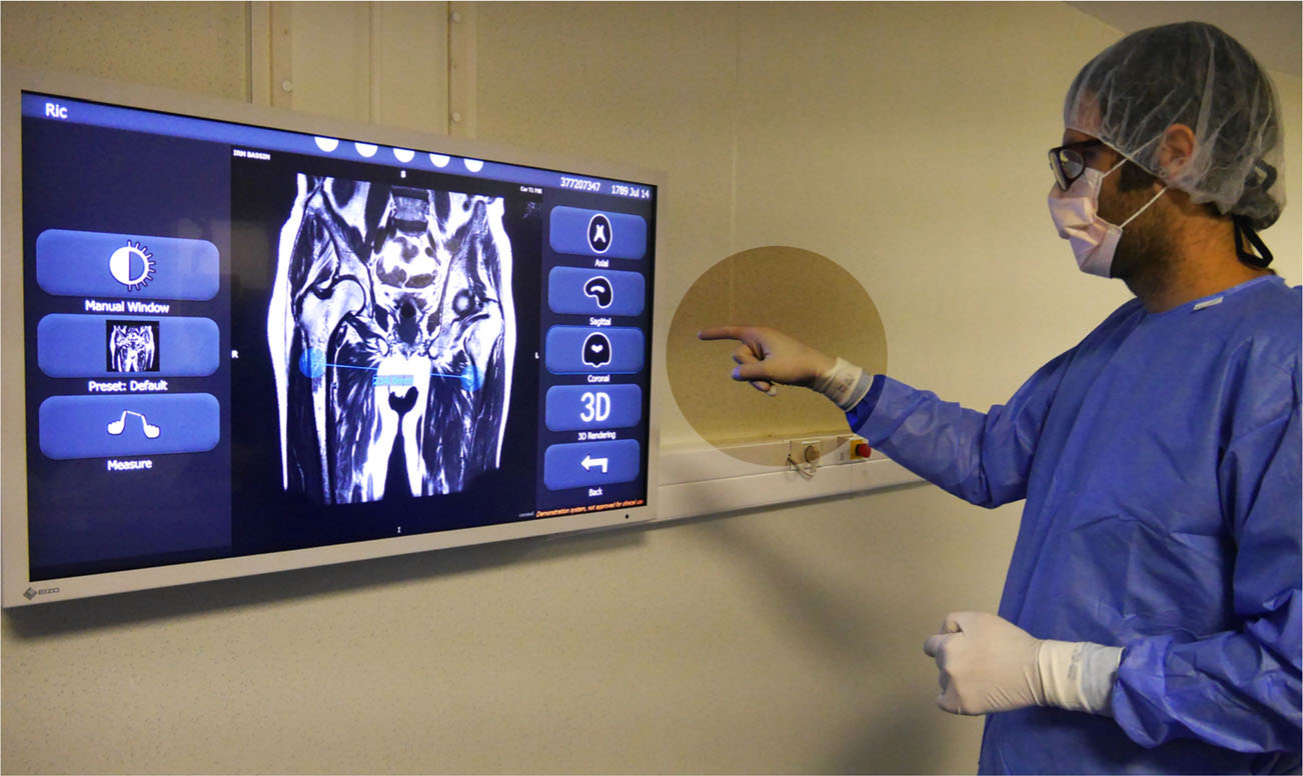
Touchless image viewer for operating rooms, Fluid (Therapixel, Paris, France). The surgeon or the interventional radiologist interacts in sterile conditions with gloved hands. The viewer interface is redesigned without a pointer; the tools are selected with lateral movements

Iannessi et al. developed and tested a touchless UI for interventional radiology [15]. Unlike previous efforts, the authors worked on redesigning a specific IU adapted to the kinetic recognition sensor without a pointer (Fig. 7). The user experience has been clearly improved with respect to simple control of the mouse pointer [16]. This is also a good example of environment constraints and user-centred design. Indeed, the amplitude of the arm movements had to be reduced to a minimum, considering the high risk of contamination inside a narrow operating room.

#### Interfaces for 3D images

Three-dimensional imaging volumes began to be routinely produced in the 1990s. They were originally acquired on MRI or reconstructed from multi-slice helical CT acquisitions, and volume acquisition later became available from rotational angiography or ultrasound as well [17]. With the exception of basic X-ray study, medical imaging examination rarely does not include 3D images.

Volume acquisition can now be printed in three dimensions, similar to the case with 2D medical films [18]. Obviously, this option can be considered only for selected cases such as preoperative planning, prosthesis or education. It is expensive and absolutely not conducive to productive workflow [19].

Some authors dispute the added value of 3D representations. Indeed, radiology explores the inside of organs, and except in rare situations, 2D slices give more information than a 3D representation of the surfaces.

**Fig. 8:**
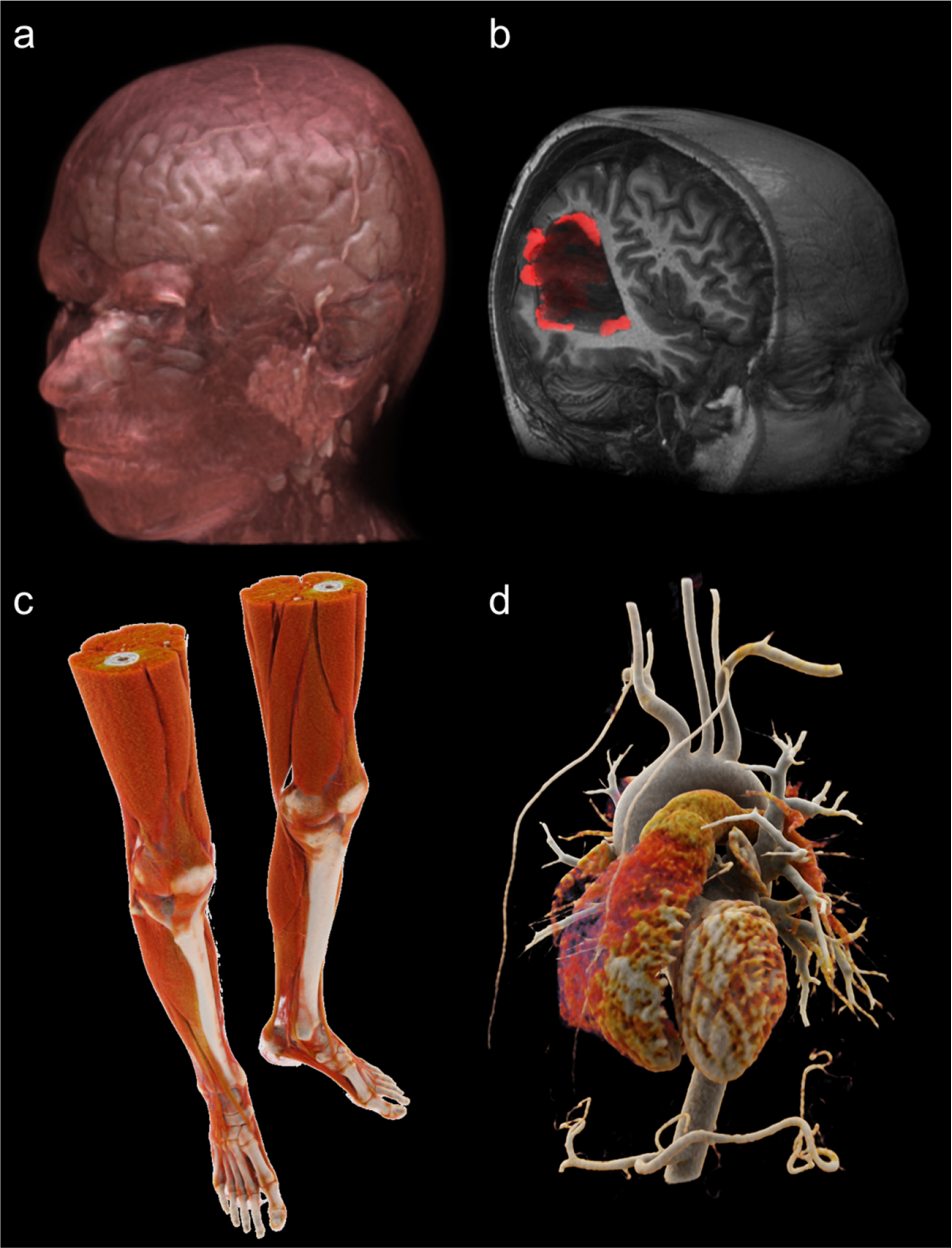
Current possibility for 3D volume rendering (VR). VR post-processed from MRI acquisition (**a**, **b**). Hyper-realistic cinematic VR processed from CT scan acquisition (**c**, **d**)

However, mental transformation from 2D to 3D can be difficult. For example, when scoliosis needs to be understood and measured, 3D UI appears to be more efficient [20]. Some orthopedic visualization, cardiovascular diagnoses and virtual colonoscopic evaluations are also improved by 3D UI [21–23]. For the same reasons, 3D volume representations are appreciated by surgeons and interventional radiologists, as they help to guide complex surgery or endovascular procedures [24–26]. Preoperative images improve surgical success [27]. Moreover, advanced volume rendering provides more realistic representations, transforming medical images into a powerful communication tool with patients (Fig. 8) [28, 29].

However, both use and usability of such acquisition volumes remain poor. There are many reasons for the non-use of 3D images, including the absence of full automation of the required post-treatment. Also, exploitation of 3D volume is hindered by the lack of adapted display and command UI [30]. By displaying 3D images on 2D screens, we lose part of the added information provided by the 3D volume [31].

With regard to inputs, touchless interfaces have been demonstrated as one interesting option. A kinetic sensor placed in front of a screen senses 3D directional movements in order to manipulate the virtual object with almost natural gestures [14, 32].

**Fig. 9:**
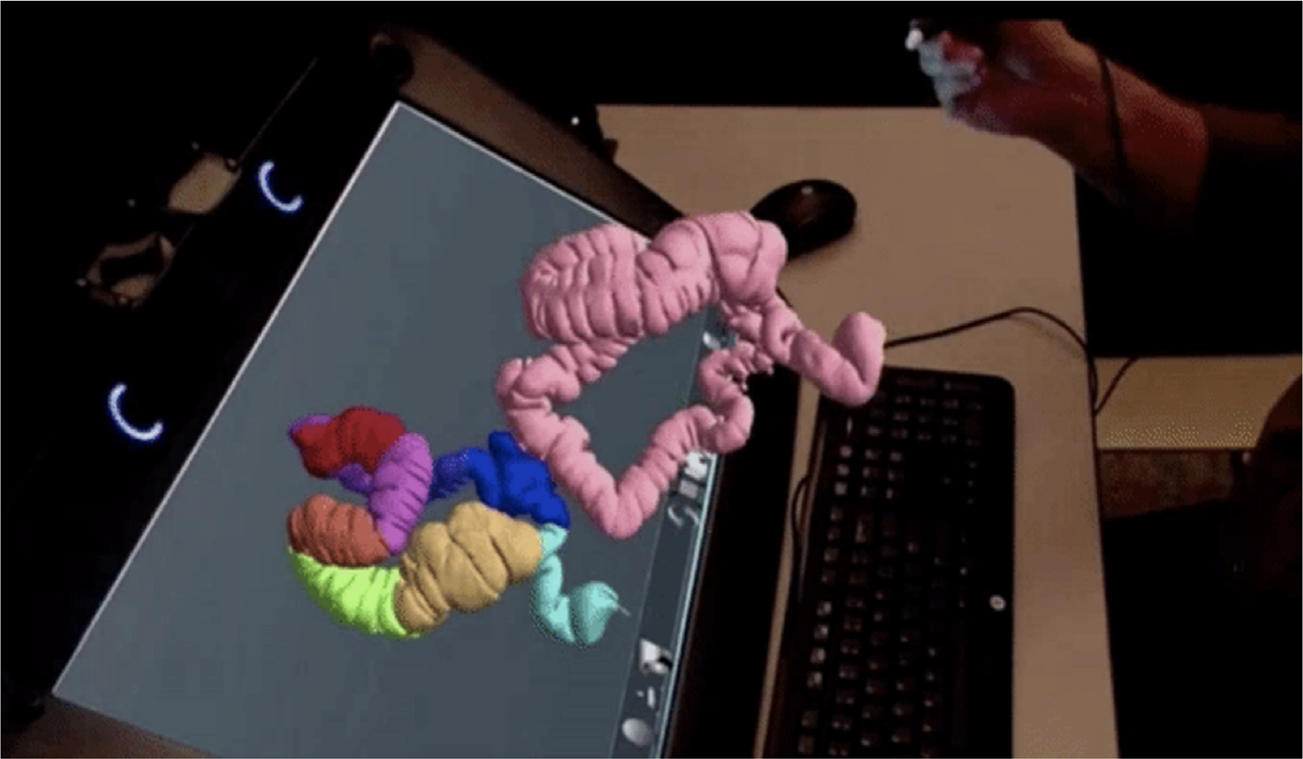
True 3D viewer (EchoPixel, Mountain View, CA, USA). This is the first holographic imaging viewer approved by the FDA as a tool for diagnosis as well as surgical planning. A stylet can interact with the displayed volume of the colon, providing an accurate three-dimensional representation of patient anatomy

For displays, some authors have explored the use of holographic imaging in radiology, especially in the field of orthopedic diagnostic imaging [23, 33, 34]. In 2015, the first holographic medical display received FDA approval. This includes 3D glasses and a stylet for manipulation (Fig. 9).

**Fig. 10:**
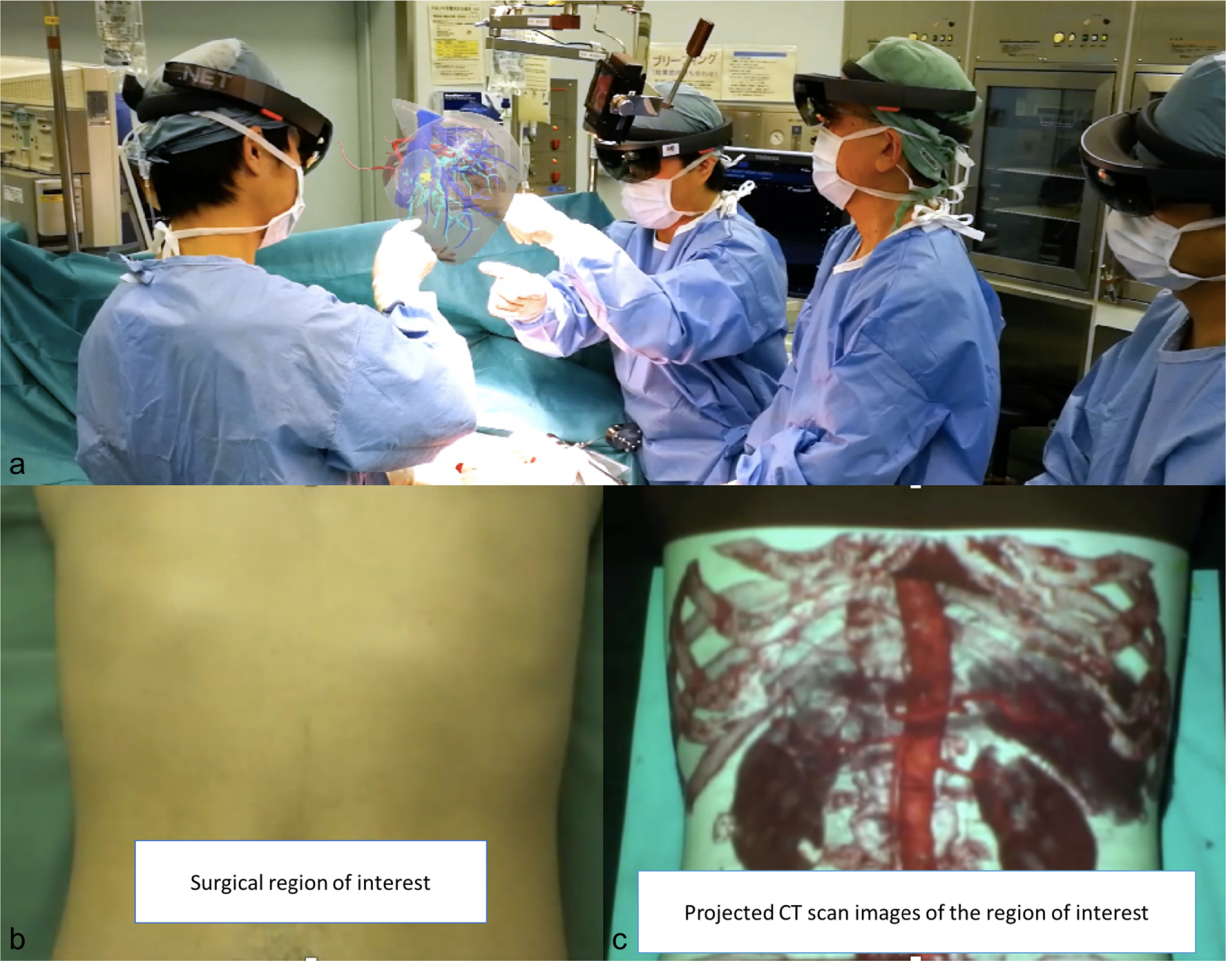
Augmented reality using 3D medical images is employed for planning and guiding surgical procedures. Surgeons wear a head-mounted optical device to create augmented reality (**a**). They can interact with the volume of the patient’s liver during surgery. Spatial augmented reality obtained by projection of the volume rendering on the patient (**b**, **c**). This see-thru visualization helps in guiding surgery

Another possibility for displaying 3D volume is the use of augmented reality. The principle is to show the images with a real-time adjustment to observe cephalogyric motion. This can be done using a head-mounted device such as Google Glass, a handheld device such as a smartphone, or a fixed device. Nakata et al. studied the latest developments in 3D medical imaging manipulation. The authors demonstrated improved efficiency of such UI compared to a two-button mouse interaction [35]. Augmented reality and 3D images have also been used in surgical practice for image navigation [36, 37]. Conventional registration requires a specific acquisition, and the process is time-consuming [38]. Sugimoto et al. proposed a marker-less surface registration which may improve the user experience and encourage the use of 3D medical images (Fig. 10) [39]. Recent promotion of the HoloLens (Microsoft, Redmond, WA, USA), a headset mixed-reality device including an efficient UI controlled by voice, eye and gesture, may help to accelerate radiological applications of augmented reality, especially for surgery (Fig. 10) [40].

**Fig. 11:**
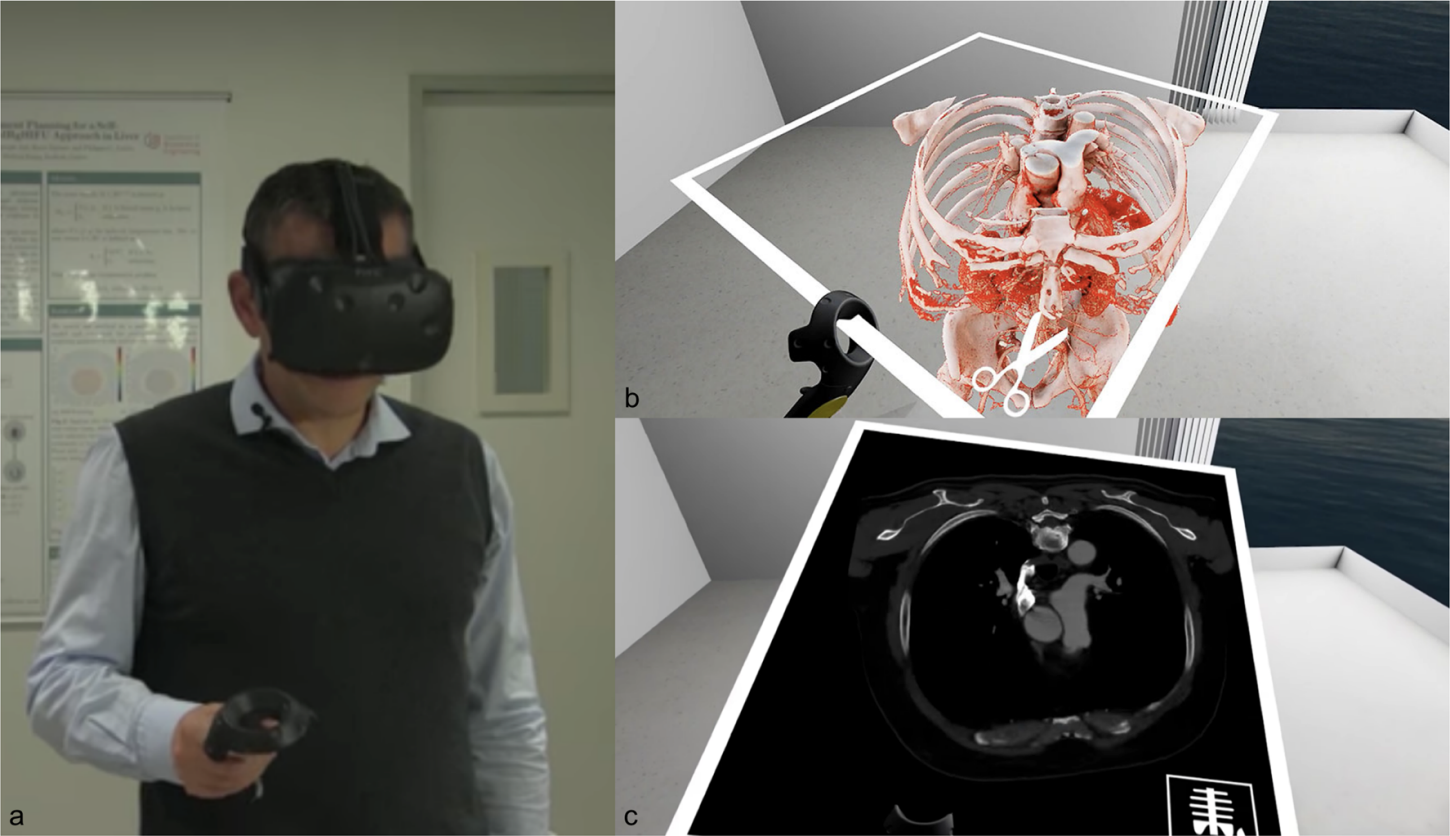
Virtual reality headset with medical images. The user wears a head-mounted display that immerses him in the simulated environments. A hand device allows him to interact with the virtual objects (**a**). The user experiences a first-person view inside and interacts with the 3D volume of the medical images (**a**). The volume can be sliced as a CT scan in any reformatted axis (**b**)

Another UI for displaying 3D medical images is virtual reality. In this case, it is a completely immersive experience. The operator wears a device and the environment is artificially created around him. Some authors have proposed including a 3D imaging volume inside the environment to give the user the opportunity to interact with it (Fig. 11).

#### Outlook for the future

**Fig. 12:**
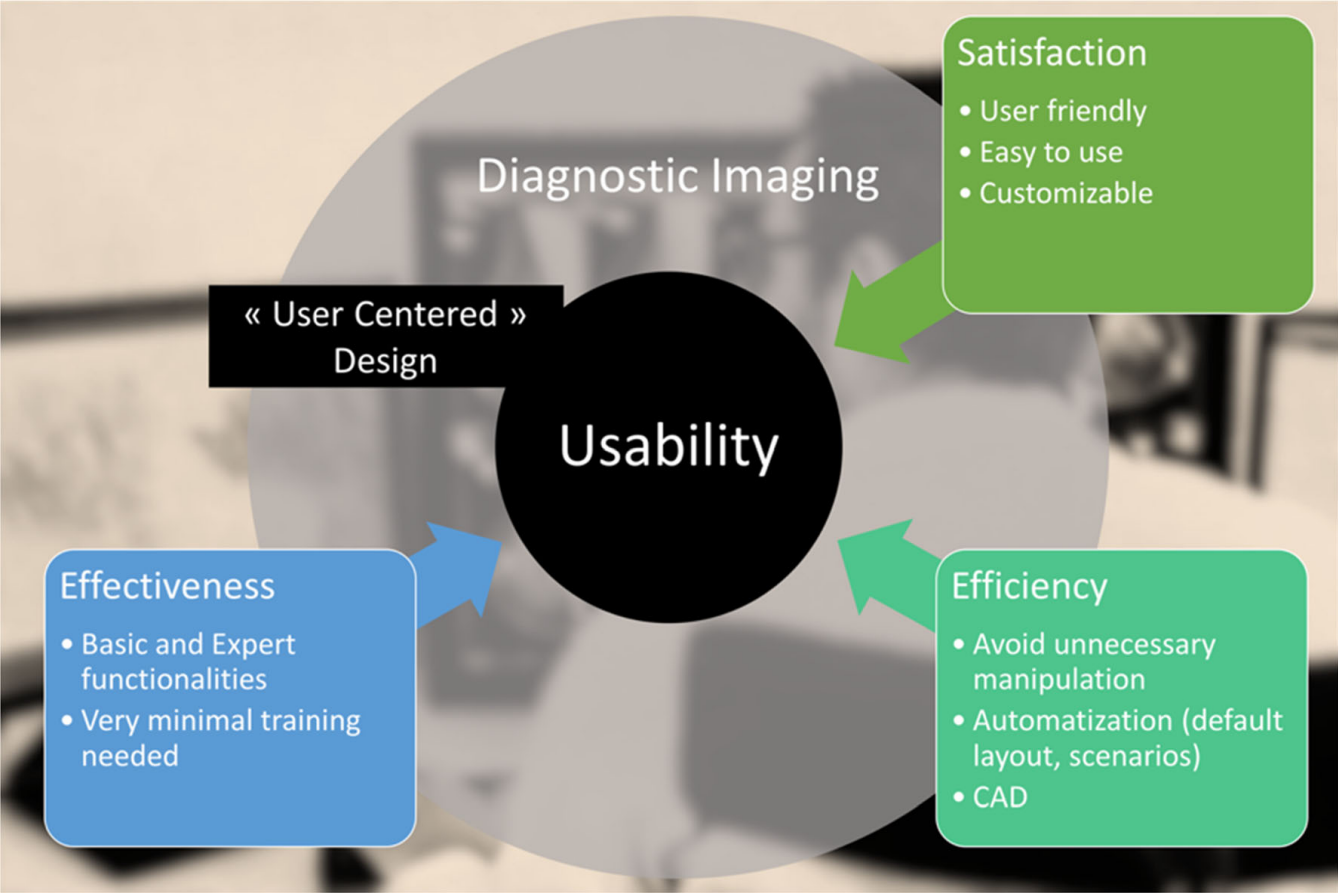
User-centred design for diagnostic imaging viewer. To maximize usability, the design of the interface needs to integrate the user and environmental requirements and constraints. Automatization and CAD have to facilitate use in order to minimize time and effort for image analysis

We believe that UX and UI specifically designed for radiology is the key for future use and adoption of new computer interface devices. A recent survey including 336 radiologists revealed that almost one-third of the radiologists were dissatisfied with their computing workflow and setup [41]. In addition to innovative hardware devices, efforts should focus on an efficient software interface. We are mainly concerned with PACS software in this discussion. Indeed, a powerful specific UI has to meet the radiologist's needs, and these needs are high (Fig. 12).

Regarding image manipulation, Digital Imaging and Communications in Medicine (DICOM) viewers are typically built with two blocks: the browser for study images and series selection, and the image viewer with manipulation tools. The key elements of the PACS UI architecture are hanging protocol and icons, image manipulation, computer-aided diagnosis and visualization features [42]. The goal of a hanging protocol is to present specific types of studies in a consistent manner and to reduce the number of manual image ordering adjustments performed by the radiologist [43]. In fact, automated scenarios should be promoted in order to present the maximum information by default at initial presentation [44]. In addition, the hanging protocols and icons should be user-friendly, intuitive and customizable. Visualization features can be incorporated into a stand-alone facility and integrated with the workstation. The software requires expert functionality that entails more than just simple scrolling, magnification and windowing.

For diagnostic imaging, in addition to the UI for image manipulation, radiologists need a UI for workflow management that includes medical records and worklists [42]. As teleradiology evolves, the concept of “SuperPACS” will probably drive the next UI to an integrated imaging viewer [45]. Indeed, medical information is tedious and labor-intensive when it is not integrated on the same interface and/or computer. The interface should aggregate all needed information for the reporting task. It is the same for the reporting and the scheduling systems. Enhancing the performance of automated voice recognition should enable real-time dictation, where we can fully interact with the images [41].

As explained above, 3D manipulation and display must be promoted for the added value they provide. Even though the technology may be ready for robust utilization, there is an intractable delay in radiologist adoption [7]. Radiologists, like any customer, are resistant to change, and the design of radiology-specific UI will hasten the revolution [14, 30].

## Conclusion

Since the digitalization of radiology, UI for radiologists have followed the evolution of common interfaces in computer science. The mouse and the keyboard remain the most widely adopted UI.

We highlight the importance of designing a specific UI dedicated to the radiologist in terms of both the hardware and software in order to make the experience more efficient, especially with the evolution of teleradiology and the need for increased productivity. Touch technology (touch or stylus) is promising, but requires exact customization for good radiologist usability.

Algorithmic advances will facilitate the take-up of 3D imaging through automated detailed and informative volume rendering. However, specific UI will be needed for 3D image display. Augmented and virtual reality are promising candidates to fill this gap. With regard to image manipulation, contactless interfaces appear to be more suitable for interventional radiology units that already have a good level of usability.
